# Cross-Protection against Acute *Staphylococcus aureus* Lung Infection in Mice by a D-Glutamate Auxotrophic Vaccine Candidate

**DOI:** 10.3390/vaccines11020210

**Published:** 2023-01-17

**Authors:** Patricia García, Maria P. Cabral, Alejandro Beceiro, Miriam Moscoso, Germán Bou

**Affiliations:** 1 Department of Microbiology, University Hospital A Coruña (CHUAC), Biomedical Research Institute A Coruña (INIBIC), 15006 A Coruña, Spain; 2 Technical Services Department, CZ Vaccines S.A.U., 36400 O Porriño, Spain; 3 Centro de Investigación Biomédica en Red de Enfermedades Infecciosas (CIBERINFEC), Instituto de Salud Carlos III, 28029 Madrid, Spain

**Keywords:** live-attenuated vaccine, *Staphylococcus aureus*, auxotrophic, intranasal immunization, glutamate racemase gene, D-amino acid transaminase gene, acute lung infection model

## Abstract

*Staphylococcus aureus* is regarded as a threatening bacterial pathogen causing invasive pneumonia in healthcare settings and in the community. The continuous emergence of multidrug resistant strains is narrowing the treatment options for these infections. The development of an effective *S. aureus* vaccine is, therefore, a global priority. We have previously developed a vaccine candidate, 132 Δ*murI* Δ*dat*, which is auxotrophic for D-glutamate, and protects against sepsis caused by *S. aureus*. In the present study, we explored the potential of this vaccine candidate to prevent staphylococcal pneumonia, by using an acute lung infection model in BALB/c mice. Intranasal inoculation of the vaccine strain yielded transitory colonization of the lung tissue, stimulated production of relevant serum IgG and secretory IgA antibodies in the lung and distal vaginal mucosa and conferred cross-protection to acute pneumonia caused by clinically important *S. aureus* strains. Although these findings are promising, additional research is needed to minimize dose-dependent toxicity for safer intranasal immunization with this vaccine candidate.

## 1. Introduction

*Staphylococcus aureus* is a versatile pathogen regarded as a leading cause of a wide range of human infections, including pneumonia [1]. It is also a commensal bacterium that colonizes about 30% of the population, generally in the skin and nares, from where it can spread to the lung. Staphylococcal pneumonia is well-documented in the hospital setting and includes health-care-associated pneumonia (HCAP), hospital-associated pneumonia (HAP) and ventilator-associated pneumonia (VAP), which are associated with high rates of morbidity and mortality [2]. It may also arise as a secondary infection from other infective foci of bacteria and as a co-infection with pneumonia caused by the influenza virus. *S. aureus* is often isolated from the airways of cystic fibrosis (CF) patients, and the elderly are at high risk of infection [3]. Community-acquired pneumonia (CAP) caused by *S. aureus* has been also documented, and can lead to complications such as severe necrotizing pneumonia, bacteremia or sepsis [4]. An association between community-acquired methicillin resistant *S. aureus* strains (CA-MRSA) carrying the gene for the Panton-Valentine leukocidin (PVL) and highly lethal necrotizing pneumonia in healthy children and immunocompetent young adults has also been reported [5].

*S. aureus* is notorious for its ability to become resistant to antibiotics, which hampers the treatment and management of associated infections [6]. Therefore, the development of alternative therapies such as vaccines is now considered a global priority for this pathogen. So far, a variety of vaccine prototypes have been explored in relation to *S. aureus* infections and some have reached clinical trials [7]. However, despite years of extensive research, a working vaccine remains elusive, highlighting the complexity of vaccination against *S. aureus*. 

We have previously developed a live vaccine containing a D-glutamate auxotrophic strain. This mutant derivative has been constructed to successfully attenuate virulence in *S. aureus* strain 132 by deleting the *murI* and *dat* genes encoding the glutamate racemase (EC 5.1.1.3) and the D-amino acid transaminase (EC 2.6.1.21) enzymes, respectively, which provide the D-glutamate required for peptidoglycan biosynthesis in this pathogen. The vaccine candidate was found to be safe and effective against systemic infection after parenteral inoculation [8]. Here, we evaluated the use of intranasal (IN) inoculation with 132 Δ*murI* Δ*dat* for preventing low respiratory infections caused by *S. aureus* and the associated mortality in mice.

## 2. Materials and Methods

### 2.1. Bacterial Strains and Culture Conditions

All bacterial strains used in this study (Table 1) were grown in Tryptic Soy Broth (TSB) or on TSB agar at 37 °C. When indicated, D-glutamate was added at 10 mM. Previously, *S. aureus* 132 Δ*murI* Δ*dat* has been constructed using an allelic exchange system with pMAD plasmid and has been genetically and phenotypically characterized [8].

### 2.2. Ethics Statement

Animal experiments were performed in full compliance with the guidelines established by the European Union (Directive 2010/63/EU) and with the national legislation (RD 53/2013). Animals were bred and maintained under specific pathogen-free conditions in the Centro Tecnológico de Formación de la Xerencia de Xestión Integrada A Coruña (CFT-XXIAC), and were provided with free access to food and water. Female BALB/c mice aged 6 to 8 weeks were used in the study.

### 2.3. Inoculation with S. aureus 132 ΔmurI Δdat and Mice Sampling

*S. aureus* 132 Δ*murI* Δ*dat* was grown in TSB plus D-glutamate with shaking until reaching an optical density measured at 600 nm of 0.7. Bacteria were collected by centrifugation, washed twice and adjusted with 0.9% NaCl (saline solution) to 10^7^, 10^8^ or 10^9^ colony forming units (CFUs) per 20 µL. Mice were anaesthetized with sevoflurane and further inoculated by the IN route with a total volume of 20 µL of bacterial suspension (10 µL to each nostril). Afterwards, mice were kept lying face up for 2 min before being removed from the anaesthetic chamber. Control mice were inoculated with saline solution in the same manner. In vivo clearance of 132 Δ*murI* Δ*dat* was evaluated for three days after IN inoculation. Blood samples were obtained by cardiac puncture from terminally anaesthetized mice, collected into EDTA tubes to avoid coagulation, and 10^−2^ serial dilutions were plated on TSB plus D-glutamate agar for enumeration of CFUs after incubation 24 h at 37 °C. Lungs and livers were aseptically removed, homogenized in sterile saline solution and the CFUs were enumerated as indicated before for blood samples. Immunization schedules consisted of IN administration of two or three doses at the indicated intervals, and mice were monitored daily to register body weight and food intake for evaluating vaccine safety. In detail, *n* = 12 mice were assigned to each experimental condition (vehicle, 10^7^, 10^8^ or 10^9^ CFUs). Mice were housed in cages of *n* = 6. Body weights were registered individually. For food intake measurements, manual weighing of food was performed for each cage every 24 h. Food intake was calculated for each cage, adjusted for g of mice body weight and expressed as mean ± S.D. for each experimental condition. Blood samples were recovered by submandibular vein puncture, and sera were obtained by centrifugation. Vaginal lavage fluid (VLF) was obtained by washing the vaginas twice with 50 µL of sterile saline solution and recovering clear supernatants in protease inhibitors (Protease Inhibitor Cocktail, Sigma-Aldrich, Merck Life Science S.L.U., Madrid, Spain) by centrifugation. Bronchoalveolar lavage was performed by insertion of a catheter in the trachea of terminally anaesthetized mice, through which a maximum of 8 mL of sterile saline solution was instilled. Several lavages were sequentially performed by injecting aliquots of 1 mL, and after each instillation the bronchoalveolar lavage fluid (BALF) was aspired. The procedure was repeated until a minimum of 4 mL of BALF was recovered for each mouse. All samples were stored at −50 °C until used.

### 2.4. Acute Lung Infection with Virulent S. aureus

Clinical isolates of *S. aureus* (Newman, USA300LAC, S1475 and Sa07965) were grown in TSB and bacterial suspensions were prepared as indicated for 132 Δ*murI* Δ*dat* strain (Section 2.3). The lethal dose for each strain was used to infect mice by IN inoculation (30 µL) in the acute lung infection model. Mice were monitored daily for survival up to 500 h post-infection. Lung bacterial burden was evaluated as above at 66 h or 30 days after infection with USA300LAC.

### 2.5. Elisa

The levels of specific IgA antibody in VLFs and BALFs, and the total IgG in mouse sera, were assessed by a whole-bacterial cell ELISA against isogenic 132 Δ*spa*, to prevent non-specific interactions of protein A with mouse immunoglobulins, and against clinical *S. aureus* Newman, USA300LAC, S1475 and Sa07965 strains (Table 1), in accordance with the protocol previously described for *S. aureus* [8]. The secondary antibodies used were horseradish peroxidase (HRP)-conjugated anti-mouse IgG (Sigma-Aldrich, Merck Life Science S.L.U., Madrid, Spain) or IgA (Bethyl Laboratories) diluted 1:5000 in DMEM supplemented with 10% fetal bovine serum. Antibody levels were expressed as Log_10_ 1/endpoint titer. The endpoint titer was defined as the maximum dilution having a value that exceeded the blank absorbance reading by 0.1 values.

### 2.6. Statistical Analysis

Statistical analysis was performed and graphics were generated in GraphPad Prism 6.01 (GraphPad Software Inc., San Diego, CA, USA). The following statistical tests were used to compare means between two or more groups of mice: two-way analysis of variance with post hoc Tukey test, Mann–Whitney *U* test, Unpaired *t* test and Log-rank (Mantel–Cox) test. Differences were considered statistically significant at *p* < 0.05.

## 3. Results

### 3.1. Dosage Escalation for Safety Assessment and Evaluation of Protective Efficacy 

**Figure 1:**
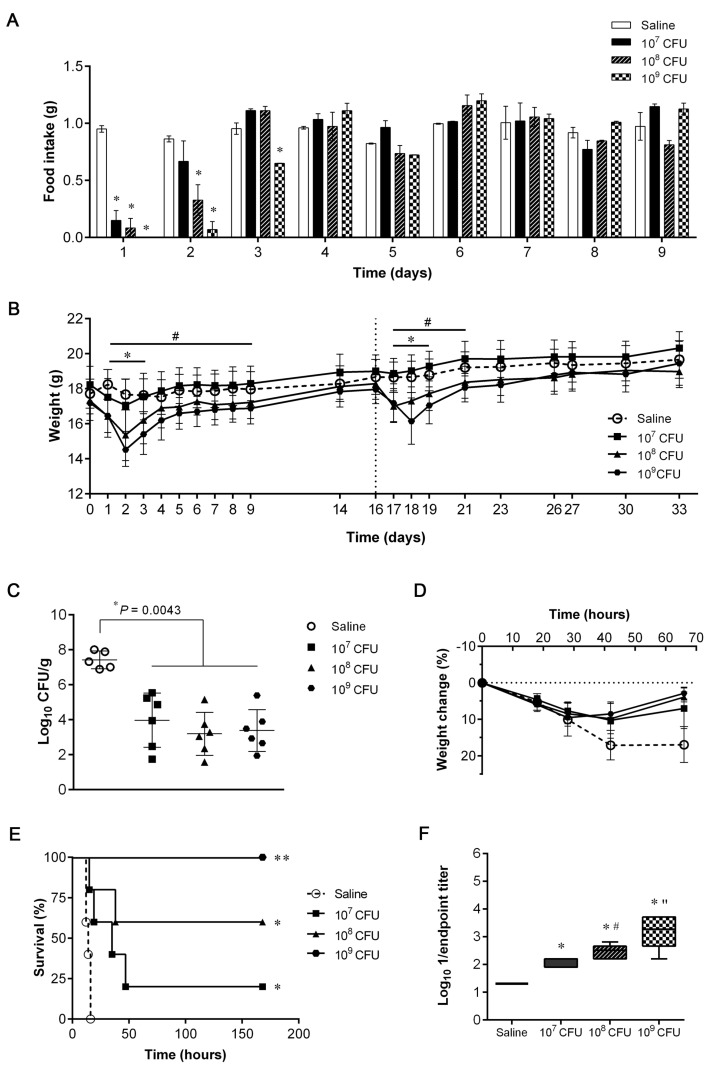
Safety, efficacy and antibody production after intranasal (IN) inoculation with escalating doses of *S. aureus* 132 Δ*murI* Δ*dat*. (**A**) Daily food intake (g/day) adjusted for g of mice body weight (*n* = 12) after IN inoculation with 132 Δ*murI* Δ*dat* (10^7^, 10^8^ or 10^9^ CFUs) or with saline. Mean ± S.D. was calculated using both cages of *n* = 6 mice from each experimental condition. * *p* < 0.05 relative to saline group, according to two-way ANOVA following by Tukey’s multiple comparisons test. (**B**) Changes in body weight of mice (*n* = 12) after IN inoculation with 132 Δ*murI* Δ*dat* (or saline) on days 0 and 16. Mean ± S.D. * *p* < 0.05 for 10^8^ CFUs and ^#^ *p* < 0.05 for 10^9^ CFUs relative to saline group, according to two-way ANOVA following Tukey’s multiple comparisons test. (**C**) Lung bacterial load of mice IN inoculated on days 0 and 16 with saline (*n* = 5) or with 132 Δ*murI* Δ*dat* (10^7^, 10^8^ or 10^9^ CFUs, *n* = 6) 66 h after IN challenge with a sub-lethal dose of USA300LAC (8 × 10^7^ CFUs) on day 34. *p*-values correspond to the Mann–Whitney *U* test. (**D**) Percentage change in body weight of mice after IN challenge and until euthanasia. Mean ± S.D. *p* = 0.0204 for 10^7^ CFUs, *p* = 0.0109 for 10^8^ CFUs and *p* = 0.0080 for 10^9^ CFUs at 66 h relative to saline, according to an unpaired *t* test with Welch’s correction. (**E**) Percentage survival of mice (*n* = 5) IN inoculated on days 0 and 14 with saline or with 132 Δ*murI* Δ*dat* (10^7^, 10^8^ or 10^9^ CFUs) after IN challenge with a lethal dose of USA300LAC (1.8 × 10^8^ CFUs) on day 21. * *p* = 0.0163 and ** *p* = 0.0025 relative to survival of mice inoculated with saline, according to Log-rank (Mantel–Cox) test. (**F**) Log_10_ 1/endpoint titer of serum IgG obtained on day 21 against 132 Δ*spa* after a two-dose immunization schedule (days 0 and 14) with saline or with 132 Δ*murI* Δ*dat* (10^7^, 10^8^ or 10^9^ CFUs). * *p* < 0.0001 relative to saline, ^#^ *p* = 0.0140 and “ *p* = 0.0350 relative to the preceding condition, according to Unpaired *t* test.

Three different escalating doses of 132 Δ*murI* Δ*dat* (10^7^, 10^8^ and 10^9^ CFUs) were used to inoculate mice (*n* = 12/group) by the IN route on days 0 and 16, or with saline (vehicle). No deaths were registered in any group. The impact of using different 132 Δ*murI* Δ*dat* inoculated CFUs was evaluated by comparing the mean food intake of immunized animals with that of the saline group (Figure 1A), which was shown to be dose-dependent.

Inoculation with 132 Δ*murI* Δ*dat* also affected the body weight of mice in a dose-dependent manner (Figure 1B). Although the 10^7^ CFU-group suffered no significant variation in body weight, inoculation with higher doses produced transient weight loss relative to mice receiving vehicle. In the short-term (within the first 72 h after inoculation), 132 Δ*murI* Δ*dat* was recovered from the lungs of inoculated mice (*n* = 4/group) as shown in Table 2. While viable bacteria were still detectable in three of four mice inoculated with 10^9^ CFUs, no CFUs were recovered from 10^7^ or 10^8^ CFU-inoculated mice at 72 h. Regardless of the dose inoculated, 132 Δ*murI* Δ*dat* bacteria were not detected beyond the lung, as no CFUs were obtained from the liver or blood at 72 h after delivery (data not shown).

After infection with a sub-lethal dose of USA300LAC, all mice receiving two doses of 132 Δ*murI* Δ*dat* showed a significant decrease in the lung bacterial burden, which was determined 66 h after infection (Figure 1C). Additionally, significant lower variations in body weight were observed for immunized mice (Figure 1D). This protective immunity was also evidenced after challenging mice with a higher dose of USA300LAC. The survival rates of 132 Δ*murI* Δ*dat*-inoculated mice were significantly higher than in the saline group, and increased with the dose (20%–60%–100%) (Figure 1E). In accordance, all immunized groups had significantly higher serum IgG levels than mice administered saline, and showed an upward trend as the dose increased (Figure 1F). Considering all these data, a concentration of 10^8^ CFUs was thus selected as the preferred dose of 132 Δ*murI* Δ*dat* for further studies.

### 3.2. Humoral Immune Responses after IN Immunization with S. aureus 132 ΔmurI Δdat

**Figure 2:**
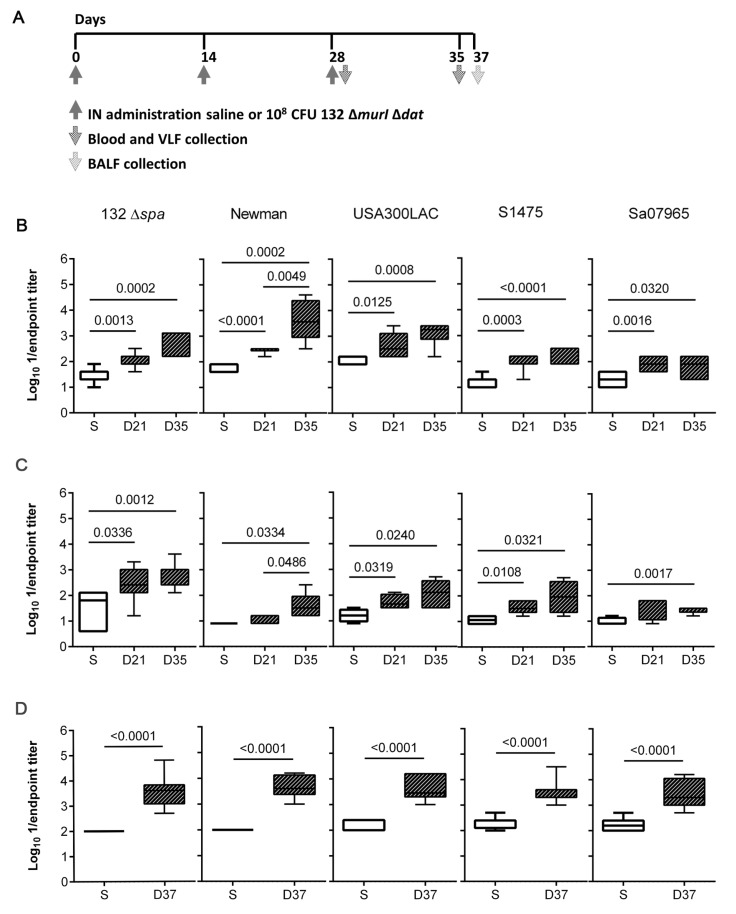
Humoral immune responses elicited after IN repeated doses of *S. aureus* 132 Δ*murI* Δ*dat*. (**A**) Diagram for timeline of immunization events and samples collection. Log_10_ 1/endpoint titer of: (**B**) serum IgG (*n* = 5–7), (**C**) vaginal IgA (*n* = 4–7), and (**D**) lung IgA (*n* = 8), obtained against the indicated strains on day 21 after two inoculations (days 0 and 14) and on days 35 and 37 after three inoculations (days 0, 14 and 28) with 10^8^ CFUs of *S. aureus* 132 ∆*murI* ∆*dat*. (**B**–**D**), *p*-values < 0.05 correspond to unpaired *t* test. VLF, vaginal lavage fluids; BALF, bronchoalveolar lavage fluid; S, saline; D, day.

The antibody levels elicited in response to IN inoculation with 132 Δ*murI* Δ*dat* were investigated after two (days 0 and 14) and three (days 0, 14 and 28) repeated doses of 10^8^ CFUs (Figure 2A). Significantly increased IgG levels were detected in sera of vaccinated mice on days 21 (after two) and 35 (after three immunizations) against the isogenic 132 Δ*spa* strain (Figure 2B). Similarly, a two-dose schedule was sufficient to detect significant IgG levels on day 21 which reacted against four heterologous *S. aureus* strains: Newman, USA300LAC, S1475 and Sa07965. Significant IgG levels were also detected after administration of three doses.

In addition, mucosal antibody responses were explored by the quantification of IgA in VLF and BALF. The vaginal IgA from mice administered with two doses of 132 Δ*murI* Δ*da*t recognized all *S. aureus* strains except Sa07965 (Figure 2C). Although the amount of vaginal IgA against Sa07965 increased significantly after the administration of three doses, the total amount remained low for this strain compared to the levels detected against the other *S. aureus* strains. Inoculation with three doses of the vaccine candidate also stimulated local production of mucosal IgA, as a significant increase in levels of IgA against all the *S. aureus* strains was observed on day 37 in BALF samples (Figure 2D).

### 3.3. Protective Efficacy Conferred by IN Immunization with S. aureus 132 ΔmurI Δdat against Acute Lung Infection Caused by Heterologous S. aureus

**Figure 3:**
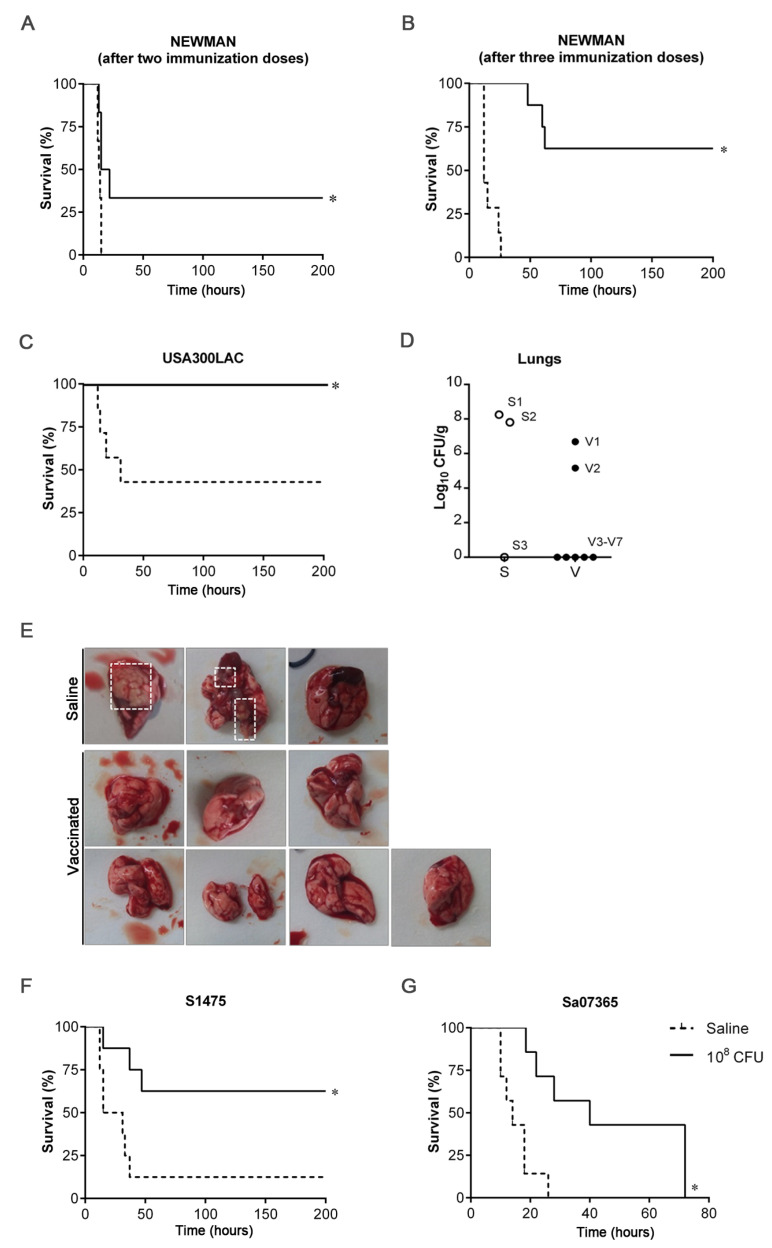
Protection efficacy against acute lung infection caused by heterologous *S. aureus* strains. (**A**) Percentage survival of mice (*n* = 6) IN inoculated on days 0 and 14 with saline or with 10^8^ CFUs of 132 Δ*murI* Δ*dat* after IN challenge with a lethal dose of Newman strain (4 × 10^8^ CFUs) on day 42. (**B**) Percentage survival of mice (*n* = 7) IN inoculated on days 0, 14 and 28 with saline or with 10^8^ CFUs of 132 Δ*murI* Δ*dat* after IN challenge with a lethal dose of Newman strain (4 × 10^8^ CFUs) on day 62. (**C**) Percentage survival of mice (*n* = 7) IN inoculated on days 0 and 14 with saline or with 10^8^ CFUs of 132 Δ*murI* Δ*dat* after IN challenge with a lethal dose of USA300LAC (2 × 10^8^ CFUs) on day 41. (**D**) Lung bacterial load from mice which had survived 30 days after infection with USA300LAC. Each symbol represents an individual mouse. (**E**) Images show lungs collected from mice represented on panel D. Dashed squares indicate surface abscesses. (**F**) Percentage survival of mice (*n* = 7) IN inoculated on days 0 and 14 with saline or with 10^8^ CFUs of 132 Δ*murI* Δ*dat* after IN challenge with a lethal dose of S1475 (1 × 10^8^ CFUs) on day 43. (**G**) Percentage survival of mice (*n* = 7) IN inoculated on days 0 and 15 with saline or with 10^8^ CFUs of 132 Δ*murI* Δ*dat* after IN challenge with a lethal dose of Sa07365 (3.5 × 10^8^ CFUs) on day 43. (**A**–**C**,**F**,**G**) * *p* < 0.05 relative to mice inoculated with saline, according to Log-rank (Mantel–Cox) test. S, Saline; V, vaccinated.

Protective efficacy against acute lung infection was evaluated (Figure 3). After challenging mice on day 42 with a lethal dose of the Newman strain, all mice administered saline succumbed to infection before 15 h, while mice vaccinated with two doses of 10^8^ CFUs of 132 Δ*murI* Δ*dat* showed 33% survival (Figure 3A). The survival rate reached 62.5% in the group administered three doses of the vaccine candidate (Figure 3B). 

After the challenge on day 41 with USA300LAC, all mice inoculated with two doses of the vaccine candidate survived. By contrast, a mortality rate of 57% was recorded in the saline group (Figure 3C). Of note, one non-immunized mouse succumbed to infection beyond 21 days after infection and was not considered for the analysis. An additional dose administrated on day 28 (a three-dose schedule) also yielded 100% survival of vaccinated mice after lethal challenge with USA300LAC (data not shown). The mice that had survived were euthanized 30 days after infection and lungs were removed (Figure 3D,E). Surface abscesses were seen in two of the three mice inoculated with saline that were compatible with the high bacterial counts detected (~8 log) in the lung tissue, but were not visible in any mice in the vaccinated group. Moreover, bacteria were not detectable in the most of these mice (five of seven).

In addition, a two-dose schedule resulted in significant higher survival of vaccinated mice (62.5%) relative to saline-inoculated mice (12.5%), all infected with S1475 (Figure 3F). Inoculation with two doses of 132 Δ*murI* Δ*dat* also extended survival after challenge with Sa07365 (Figure 3G). A three-dose schedule was not shown to improve the survival rate of inoculated mice (data not shown).

## 4. Discussion

*S. aureus* is ranked among the most prominent human pathogens of global concern and a leading cause of deaths associated with antibiotic resistance [13,14]. It causes a range of illness, including severe respiratory infections, for which no prophylactic vaccines are yet available. In addition, there are no ongoing clinical trials aimed at preventing *S. aureus*-induced pneumonia. A number of vaccine prototypes have been tested to reduce the severity of staphylococcal lung infection in animal models [15,16,17,18,19,20,21]. Most of these studies performed parenteral immunizations, mainly via intramuscular or subcutaneous routes. In this work, we explored the IN route of inoculation to evaluate the immunogenic potential and effectiveness of the D-glutamate auxotroph of *S. aureus* in a mouse model of acute lung infection.

The activation of mucosal immunity in the respiratory tract is crucial for protection against respiratory infections [22]. Thus, IN vaccination would provide benefits relative to the same formulation injected parenterally to stimulate local immune responses in nasopharynx-associated lymphoid tissue (NALT) and lungs, but also at distant sites such as the gastric mucosa and the genital tracts [23]. Accordingly, IN administration of 132 Δ*murI* Δ*dat* (two/three doses of 10^8^ CFUs) promoted increments in IgA levels that were detected locally in BALF and also at distal sites of inoculation as observed in the VLF samples from vaccinated mice. The treatment also triggered serum IgG antibodies that, along with secretory IgA, were able to recognize genotypic diverse *S. aureus* strains to a varying degree. Numerous studies suggest that antibody response should be accompanied by an appropriate vaccine-induced cellular immunity to achieve optimal protection against *S. aureus* infections [24]. In particular, the protective role of interleukin IL-17 has been extensively demonstrated against skin and systemic *S. aureus* disease, although, depending on the infection model used, IFN-γ has been also pointed out as a crucial protective cytokine [25,26,27,28,29,30,31]. However, existing evidence about the nature of protective cellular immune responses against staphylococcal pneumonia is scarce. IL-17-secreting T cells were probed to play a role in host response against *S. aureus* pneumonia and, recently, to be implicated in vaccine-induced protection against airway infection [21]. Both CD4^+^ Th17 and γδ T cells have been documented as IL-17-producers in the lung environment responsible for a protective effect [32,33], but also specific lung CD4^+^ Th1 could attenuate the severity of *S. aureus* pneumonia [34]. Interestingly, after immunization by the IN route, the production of IL-17 in the lung has been found to be associated with local IgA responses [35], and this association could be also mounted with 132 Δ*murI* Δ*dat.* In fact, IL-17-secreting splenocytes were the predominant T-cell subset triggered by parenteral 132 Δ*murI* Δ*dat* injections and, as previously noted, correlated with long-term defense against lethal systemic infection caused by this bacterium [8].

Furthermore, a cross-protective effect of IN vaccination was demonstrated by a reduction in the mouse mortality rate after acute lung infection with the Newman strain and two CA-MRSA strains. Newman is a clinical strain and well-known producer of α-hemolysin (Hla), an essential virulence factor in the pathogenesis of lung infection [36]. On the other hand, USA300LAC and S1475 strains, which represent, respectively, major USA and European clones of CA-MRSA, harbor the PVL toxin, which is associated with necrotizing pneumonia [5,37]. In particular, S1475 caused the death in 2001 of a child without prior healthcare exposure who suffered necrotizing pneumonia within 48 h of hospitalization. Moreover, IN vaccination prevented lung abscess formation in the long-term in mice surviving after infection with USA300LAC. Furthermore, some protection against Sa07365 was observed, as a notable delay in time to death of vaccinated mice was recorded. The high levels of mucosal IgA induced in lungs (as detected from BALF samples) after IN vaccination may be partly due to the ability of the live vaccine to translocate from nares to the lower airway. Fan et al. [21] revealed that reaching the deep lung seems crucial for efficacious immunity, and this could not be achieved by IN inoculation when using a subunit vaccine candidate containing staphylococcal clumping factor A (ClfA). Thus, for non-living vaccines, intrapulmonary immunization may be necessary for protective efficacy. By contrast, after IN administration, the D-glutamate auxotroph of *S. aureus* was able to migrate and transiently colonize lung tissue before elimination, which might have been the cause of enhanced protective efficacy against lung disease. In addition, this whole-cell vaccine contains all the antigenic determinants in native conformation that are expressed in vivo to be initially recognized by tissue Toll-like receptors (TLR) and to successfully induce the innate immune system for trained immunity. Moreover, triggering the TLR-associated signaling is pivotal in orchestrating the specific adaptive immunity [38]. As a consequence, TLR agonists were explored as modulatory agents for preventing *S. aureus* infection (CpG-DNA TLR9 agonist) and as adjuvants for increasing vaccine efficacy (4C-Staph formulated with TLR7-dependent molecule) [39,40]. Unfortunately, despite the promising results generated by the IN route with 132 Δ*murI* Δ*dat*, a dose-dependent toxic effect was manifested in mice, which experienced a transient decrease in food consumption and body weight. Therefore, additional studies aimed at eliminating toxic effects are essential before further contemplation of IN inoculation with this vaccine strain.

## 5. Conclusions

IN administration of the D-glutamate auxotroph of *S. aureus* elicits relevant serum IgG and mucosal IgA antibodies in mice and confers cross-protection to clinically important *S. aureus* strains in an acute lung infection model. Future research is needed to improve the safety profile for IN administration and to drive progress in developing prophylactic treatments against staphylococcal pneumonia with this vaccine candidate.

## Figures and Tables

**Table 1:** *S. aureus* strains used in this study.

Strain	Relevant Features	Reference
132	MRSA clinical isolate, PVL-positive	[9]
132 Δ*murI* Δ*dat*	132 derivative, MurI*–*, Dat–deficient	[8]
132 Δ*spa*	132 derivative, SpaA (protein A)–deficient	[9]
Newman	MSSA, isolated from human infection	[10]
FPR3757 (USA300LAC)	CA-MRSA, isolated from wrist abscess, USA300 clone ST8, PVL-positive	[11]
S1475	CA-MRSA, isolate from necrotizing pneumonia, European clone ST80, PVL-positive	[12]
Sa07365	MSSA, isolated from staphylococcal pneumonia and co-infection with influenza A virus	Laboratory Collection, CHUAC

CA-MRSA, community-acquired methicillin-resistant *S. aureus*; MSSA, methicillin-sensitive *S. aureus*; PVL, Panton-Valentine leukocidin toxin; ST, sequence type; CHUAC, University Hospital A Coruña.

**Table 2:** Lung bacterial load expressed as Log_10_ CFUs/g (mean ± S.D.) after IN inoculation with *S. aureus* 132 Δ*murI* Δ*dat*.

	Time (h) Post-Inoculation		
Dose (CFUs/Mouse)	24	48	72
10^7^	4.24 ± 0.33 (4/4) ^1^	2.30 ± 0.20 (3/4)	0.00 ± 0.00 (0/4)
10^8^	5.78 ± 0.34 (4/4)	3.62 ± 0.47 (4/4)	0.00 ± 0.00 (0/4)
10^9^	7.07 ± 0.29 (4/4)	4.27 ± 0.41 (4/4)	2.57 ± 0.31 (3/4)

^1^ Number of mice yielding positive cultures/total mice are shown in brackets.

## Data Availability

Data available upon request.
